# Yersinia pestis Exploits Early Activation of MyD88 for Growth in the Lungs during Pneumonic Plague

**DOI:** 10.1128/IAI.00757-18

**Published:** 2019-03-25

**Authors:** Rachel M. Olson, Miqdad O. Dhariwala, William J. Mitchell, Deborah M. Anderson

**Affiliations:** a Department of Veterinary Pathobiology, University of Missouri, Columbia, Missouri, USA; b Laboratory of Infectious Disease Research, University of Missouri, Columbia, Missouri, USA; Yale University School of Medicine

**Keywords:** MyD88, Toll-like receptor, Yersinia, *Yersinia pestis*, plague, pneumonia, sepsis

## Abstract

Yersinia pestis causes bubonic, pneumonic, and septicemic plague. Although no longer responsible for pandemic outbreaks, pneumonic plague continues to be a challenge for medical treatment and has been classified as a reemerging disease in some parts of the world. In the early stage of infection, inflammatory responses are believed to be suppressed by Y. pestis virulence factors in order to prevent clearance, while later, the hyperactivation of inflammation contributes to the progression of disease. In this work, we sought to identify the host factors that mediate this process and studied the role of the Toll/interleukin 1 (IL-1) receptor adapter and major inflammatory mediator myeloid differentiation primary response 88 (MyD88) in pneumonic plague. We show that pulmonary challenge of *Myd88^−/−^* mice with wild-type (WT) Y. pestis results in significant loss of pro- and anti-inflammatory cytokines and chemokines, especially gamma interferon (IFN-γ) and KC, in the lungs compared to that in WT mice. Bacterial growth in the lungs occurred more rapidly in the WT mice, however, indicating a role for the MyD88 response in facilitating the primary lung infection. Nevertheless, *Myd88^−/−^* mice were more sensitive to lethality from secondary septicemic plague. Together these findings indicate a central role for MyD88 during the biphasic inflammatory response to pulmonary Y. pestis infection. In the early phase, low-level MyD88-dependent chemokine expression limits initial growth but facilitates Y. pestis access to a protected replicative niche. The later hyperinflammatory phase is partially MyD88 dependent and ineffective in the lungs but controls systemic infection and reduces the progression of secondary septicemic plague.

## Abstract (precis)

Yersinia pestis causes bubonic, pneumonic, and septicemic plague. Although no longer responsible for pandemic outbreaks, pneumonic plague continues to be a challenge for medical treatment and has been classified as a reemerging disease in some parts of the world.

## INTRODUCTION

The activation of innate immune signaling begins with the recognition of potential pathogens by surface-located receptors, a process widely conserved from insects to humans. Conserved bacterial molecular patterns, such as lipid A and teichoic acids, are recognized by Toll-like receptors (TLRs), which assemble homo- and heterodimeric structures with the capability for binding cytoplasmic proteins, mainly myeloid differentiation primary response 88 (MyD88) (1, 2). Binding of MyD88 by dimerized TLRs results in the assembly of an oligomeric complex known as the myddosome, which transmits a molecular signal to induce the expression of inflammatory cytokines through nuclear factor kappa light chain enhancer of activated B cells (NF-κB) and other transcriptional activators (3). Binding between activated TLRs and MyD88 is mediated by their respective cytoplasmic C-terminal Toll-interleukin 1 receptor (IL-1R) (TIR) domains, which allows assembly of the myddosome. Assembly of the myddosome activates a phosphorylation cascade that leads to the inactivation of the inhibitor of NF-κB (IκB), allowing the nuclear translocation of NF-κB. Other TIR domain-containing proteins, including those of the IL-1 receptor family, also bind and activate MyD88 for the activation of NF-κB.

During bacterial infection, the production of neutrophil chemokines as well as gamma interferon (IFN-γ) is often dependent on IL-18 receptor signaling (4). Mice lacking MyD88 are highly susceptible to many bacterial and parasitic infections, indicating its central importance to innate immunity (5). Immune and endothelial cells, as well as hematopoietic stem cells, express TLRs, and the hyperactivation of MyD88 during infection is associated with hematopoietic diseases as well as a number of inflammatory disorders (6). Furthermore, the hyperactivation of expression of inflammatory cytokines during bacterial infection is associated with tissue damage and can be a significant contributor to multiorgan failure and lethality.

Plague is a lethal flea-borne infectious disease caused by the Gram-negative bacterium Yersinia pestis. A rapidly progressing infection occurs following the bite of an infected flea or the inhalation of airborne bacteria, causing death in only a few days without antibiotic intervention (7, 8). The bubonic form of plague is endemic in many areas of the world, including the southwestern United States, stabilized in the environment by a rodent-flea transmission cycle. Although humans are incidental hosts, bubonic plague cases occur each year and continue to cause mortality even with antibiotic treatment (8, 9). Primary pneumonic plague is currently the most feared form of the disease, as the recently reported mortality rates from this disease have exceeded 50% in some outbreaks. In Madagascar, where plague is endemic and human cases have increased since the beginning of the 21st century, multidrug-resistant Y. pestis strains have been isolated, indicating the continual genetic evolution of this pathogen (10–12). Natural drug resistance is believed to arise from high-efficiency horizontal gene transfer in the flea midgut (13). Genetic engineering of drug resistance or other virulence traits present the additional potential threat that the organism can be weaponized. For these reasons, there remains a pressing need to understand the pathogenesis of plague in order to develop improved treatments.

Although the hyperactivation of the host inflammatory response is thought to contribute to the rapid progression of plague, the underlying mechanisms in the host are not well understood. Several bacterial virulence factors are known to suppress the host response to infection. For example, hypoacylation of lipid A occurs when Y. pestis is growing at 37°C, and this form of lipopolysaccharide (LPS) appears to antagonize TLR4, minimizing its role in defense against infection (14, 15). Yersinia pestis bacteria engineered to express hexaacylated lipid A during mammalian infection were significantly attenuated and subject to clearance by TLR4 and the NLRP12 inflammasome, suggesting that hypoacylation of lipid A renders these host defense mechanisms useless (16, 17). Nevertheless, endotoxin purified from Y. pestis cultures is believed to contribute to the hyperactive inflammatory response and the resulting rapid lethality of plague, indicating that hypoacylation of lipid A does not neutralize its stimulation of the innate immune response (18).

In addition, the bacterial type III secretion system (T3SS) provides suppression of the inflammatory response. *Yersinia* targets phagocytic cells, especially macrophages, and upon intimate contact, the T3SS translocates virulence factors, collectively known as *Yersinia* outer proteins (Yops), into the host cell cytoplasm (19, 20). Once in the host cell cytoplasm, Yops interfere with intracellular processes, including those required for TLR signaling (21). Yops also prevent phagocytosis and modulate multiple programmed cell death pathways in a manner that likely has an impact on the inflammatory response. For example, injection of YopJ into naive bone marrow-derived macrophages has been shown to induce apoptosis, whereas activated macrophages induce inflammatory forms of cell death (22). Following pulmonary infection of mice, the primary targets of the T3SS appear to be alveolar macrophages, and this correlates with an apparent immune suppressive phase in the lungs (23, 24).

Despite the overall immune suppression provided by the T3SS, neutrophils are recruited to infected tissues. Antibody ablation of neutrophils has been shown to cause increased susceptibility of mice to pneumonic plague, indicating an overall protective role (25). Consistent with this hypothesis, mice lacking the neutrophil chemokine KC or its receptor CXCR2 are more susceptible to disease, with an increased bacterial burden in the lungs (26). These observations are counter to the expected result that cytokine and chemokine production were suppressed in the lungs by the mechanisms discussed above until late in the infection. In this work, in order to better understand these observations and the role of inflammation in pneumonic plague, we characterized pulmonary Y. pestis infection of *Myd88^−/−^* mice. These mice are unable to mobilize an NF-κB cytokine and chemokine response following TLR or IL-1R activation.

## RESULTS

### MyD88-dependent inflammation facilitates growth of Y. pestis in the lungs.

Intranasal infection of mice with wild-type Yersinia pestis CO92 results in the development of primary pneumonic plague in 3 to 4 days, characterized by focal bacterial growth and neutrophil recruitment to the alveoli, as well as vascular dissemination and the development of secondary septicemic plague, with high levels of serum cytokines and multiorgan failure (27). Secondary tissues, including the liver and spleen, subsequently become heavily colonized, a condition that likely contributes to lethality. Therefore, as it does in humans, pulmonary infection of mice with Y. pestis leads to the development of two lethal diseases: bronchopneumonia and sepsis.

**FIG 1:**
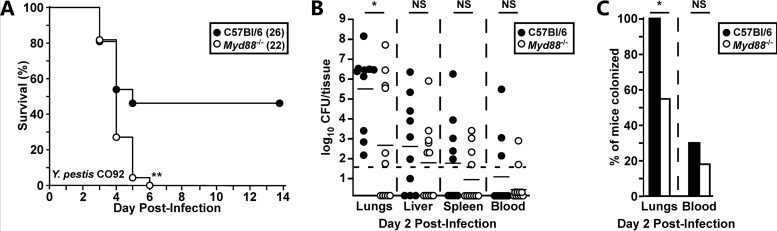
MyD88 contributes to growth of wild-type Y. pestis in the lungs. Groups of 5 to 10 C57BL/6 (filled circles or bars) or *Myd88^−/−^* (open circles or bars) mice were challenged by intranasal infection with 2,000 CFU of Y. pestis CO92. (A) Survival was monitored over 14 days, data were collected in 3 independent trials, and the total number analyzed for each group is shown in parentheses; data were combined and analyzed by the Mantel-Cox log rank test. **, *P* < 0.01. (B and C) On day 2 postinfection, mice were euthanized, tissues and blood were collected, and bacterial titers in the lungs, liver, spleen, and blood were determined (B) and are shown as percent colonization (C). Data were collected in 2 independent trials; *n* = 10 WT mice, *n* = 11 *Myd88^−/−^* mice. Bars in panel B represent the median; bars in panel C represent the standard error. Combined data were analyzed by Mann-Whitney (B) and chi-square (C) tests, with each tissue analyzed independently. *, *P* < 0.05; NS, not significant.

To characterize the role of MyD88 in primary pneumonic plague, we challenged wild-type C57BL/6 (WT) and *Myd88*^−/−^ mice by intranasal infection with Yersinia pestis CO92. At a low challenge dose (1 to 3 50% lethal doses [LD_50_]), 50% of WT mice survived the infection, whereas the *Myd88*^−/−^ mice were significantly more susceptible and succumbed on days 3 and 4 postinfection (Fig. 1A). To determine if MyD88 was required for controlling bacterial growth in the lungs, we quantified the number of Y. pestis CFU (per organ) at 48 h postinfection (hpi). Unexpectedly, *Myd88^−/−^* mice had significantly reduced bacterial growth in the lungs compared to WT mice at 48 hpi (Fig. 1B). In fact, there was a significant increase in the number of mice with no detectable lung bacterial titer when MyD88 was absent, indicating that MyD88 facilitates the growth of Y. pestis in the lungs of WT mice (Fig. 1C). Bacterial growth levels in the liver, spleen, and blood appeared to be similar between WT and *Myd88^−/−^* mice, suggesting that the MyD88 response does not substantially affect the dissemination and growth of bacteria in the secondary tissues.

**FIG 2:**
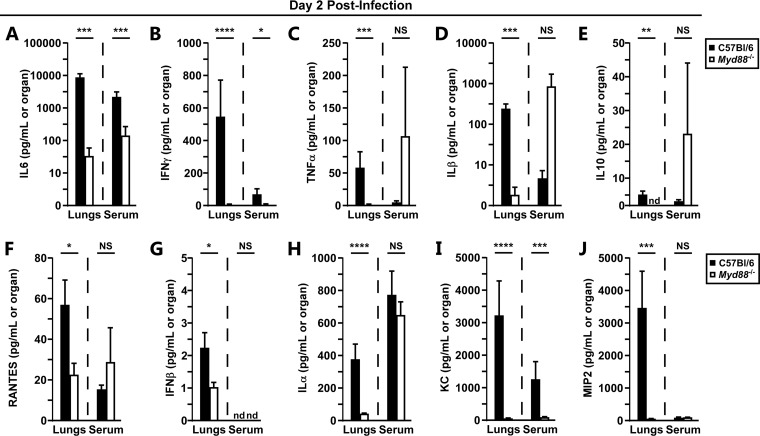
Myd88 controls early pulmonary cytokine responses to Y. pestis. Groups of 5 to 6 C57BL/6 (filled bars) or *Myd88^−/−^* (open bars) mice were challenged by intranasal infection with 2,000 CFU of Y. pestis CO92. On day 2 postinfection, mice were euthanized, and lungs and blood were collected for analysis of the following cytokines: IL-6 (A), IFN-γ (B), TNF-α (C), IL-1β (D), IL-10 (E), RANTES (F), IFN-β (G), IL-1α (H), KC (I), and MIP2 (J). Data were collected in 2 independent trials; *n* = 10 WT mice; *n* = 11 *Myd88^−/−^* mice. Bars represent standard errors. nd, not detected. Combined data were analyzed by the Mann-Whitney test, with lungs and serum analyzed independently. *, *P* < 0.05; **, *P* < 0.01; ***, *P* < 0.001; ****, *P* < 0.0001; NS, not significant.

We measured NF-κB-regulated cytokines and chemokines, including IL-1, IL-6, tumor necrosis factor alpha (TNF-α), IL-10, IFN-β, IFN-γ, KC, and others, in infected lung homogenates at 48 hpi. Consistent with the reduced bacterial burden, the lungs of *Myd88^−/−^* mice harbored significantly reduced levels of all 10 of the cytokines and chemokines we measured (Fig. 2A to J). In fact, even those *Myd88^−/−^* mice in which high bacterial titers were recovered had low levels of inflammatory cytokines. This indicates that the production of cytokines in the lungs is heavily dependent on MyD88 during the early stage of infection. In striking contrast, however, many inflammatory cytokines, TNF-α, IL-1α, IL-1β, IL-10, RANTES, and MIP-2, were elevated in the sera of both groups of mice at 48 hpi, with no detectable differences in the median serum concentrations between groups, indicating that the onset of secondary sepsis is likely independent of MyD88 (Fig. 2B, F, H to J). Serum IL-6, IFN-γ, and KC, however, were dependent on MyD88, whereas serum IFN-β was undetectable in both groups of mice at 48 hpi (Fig. 2A, B, G to I). These data indicate that serum cytokines are produced through MyD88-independent and -dependent mechanisms.

### The biphasic inflammatory response is MyD88 dependent.

**FIG 3:**
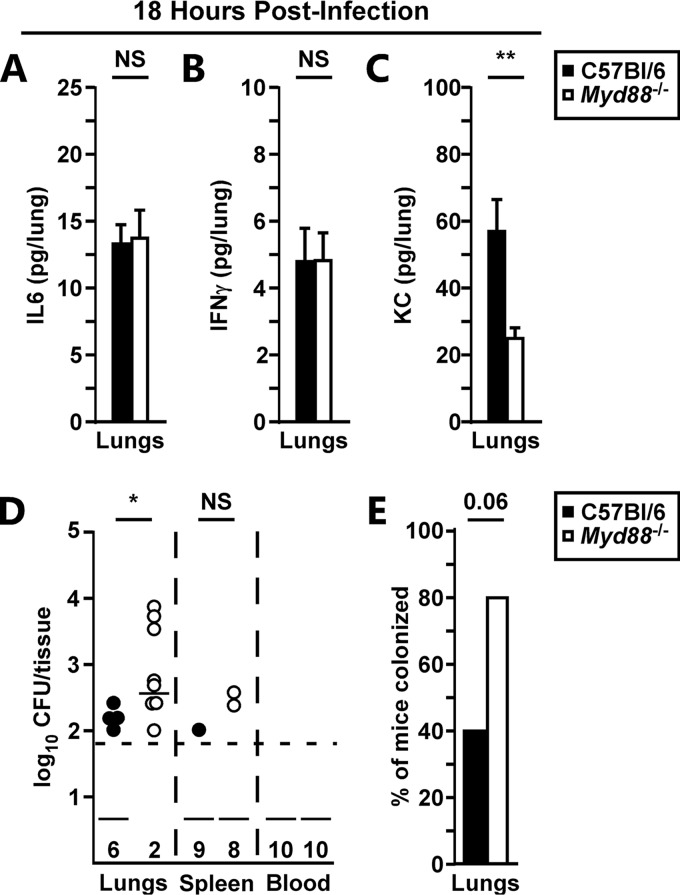
Early proinflammatory cytokines and colonization of the lungs are MyD88 dependent. Groups of 5 C57BL/6 (filled bars) or *Myd88^−/−^* (open bars) mice were challenged by intranasal infection with 2,000 CFU of Y. pestis CO92. (A to D) After 18 h of infection, the mice were euthanized, and lungs were collected for cytokine and CFU analyses: IL-6 (A), IFN-γ (B), KC (C), and CFU (D). (E) Representation of mouse colonization from panel D data. Data were collected in 2 independent trials; *n* = 10. Bars represent standard errors. Combined data were analyzed by an unpaired *t* test (A to C), the Mann-Whitney test (D), or the chi-square test (E). *, *P* < 0.05; **, *P* < 0.01; NS, not significant.

To determine how early MyD88 activation was important to the host response in the lungs, we measured lung cytokines at 18 hpi. In contrast to the findings at 48 hpi, most of the inflammatory cytokines, including IL-6 and IFN-γ, were suppressed in the lungs at 18 hpi, consistent with the immune suppressive phase of the infection (Fig. 3A and B; see Fig. S1A to H in the supplemental material). However, the chemokine KC was MyD88 dependent, with a significant reduction in KC levels found in *Myd88^−/−^* mice at 18 hpi (Fig. 3C). To determine if this had an impact on bacterial growth, we also measured lung bacterial titers at 18 hpi. Once again in contrast to the 48 hpi time point, we recovered significantly higher bacterial titers in the lungs of *Myd88^−/−^* mice at 18 hpi (Fig. 3D). Together these data suggest that MyD88-dependent KC may be responsible for an early protective immune response. Furthermore, these data suggest that both phases of the biphasic inflammatory response to pulmonary infection with Y. pestis are MyD88 dependent.

### Loss of MyD88 accelerates the progression of secondary sepsis.

**FIG 4:**
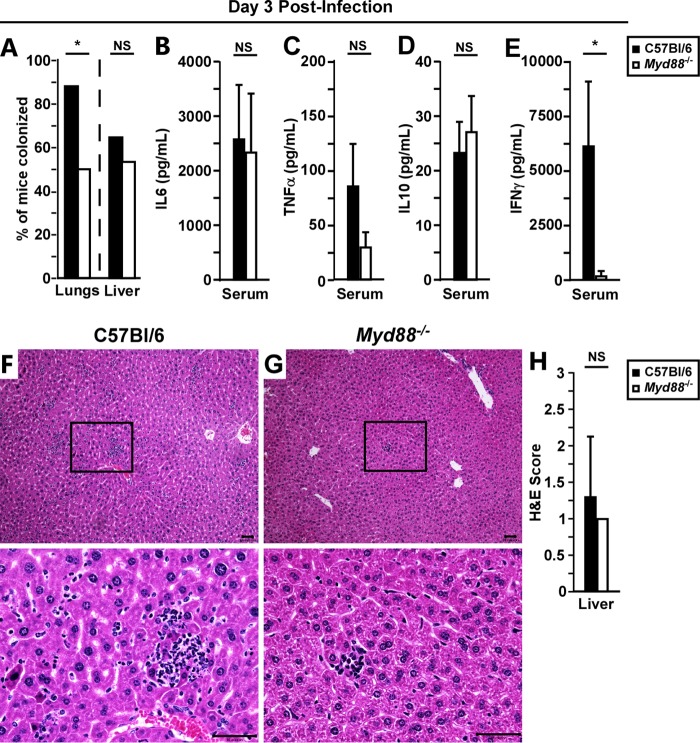
Peripheral responses to Y. pestis are MyD88 independent. Groups of WT or *Myd88^−/−^* mice were challenged by intranasal infection with 2,000 CFU of Y. pestis CO92. On day 3 postinfection, the mice were euthanized, and blood and tissues were removed for further processing. (A) Percentage of mice with bacterial colonization of lungs or liver. (B to E) Sera were analyzed for cytokines IL-6 (B), TNF-α (C), IL-10 (D), and IFN-γ (E). (F to H) Representative liver histopathology (F and G) and quantification of lesion severity (H) for WT (F) and *Myd88^−/−^* (G) mice at 72 hpi. Scale bar, 50 μm. Boxes in top panels indicate the zoomed-in sections shown in the bottom panels. Bars represent standard deviations. ND, not detected. Combined data from panel A (*n* = 17 WT mice, *n* = 12 *Myd88^−/−^* mice; collected in 2 independent trials) were analyzed by the chi-square test. Combined data from panels B, C, and E (*n* = 40 WT mice, *n* = 36 *Myd88^−/−^* mice; collected in 4 independent trials) and panel D (*n* = 30 WT mice, *n* = 26 *Myd88^−/−^* mice; collected in 2 independent trials) were analyzed by the Mann-Whitney test; combined data from panel H (*n* = 10 per group; collected in 2 independent trials) were analyzed by an unpaired Student’s *t* test. *, *P* < 0.05; NS, not significant.

To better understand why *Myd88^−/−^* mice were more sensitive to lethality, we examined local and disseminated infection at 72 hpi. In agreement with the findings at 48 hpi, significantly more *Myd88^−/−^* mice harbored undetectable bacterial titers in the lungs at 72 hpi (Fig. 4A). In contrast, liver colonization rates were similar between mouse groups, suggesting little to no impact of MyD88 at this stage in controlling bacterial growth in the secondary infected tissues. Similar to the findings at 48 hpi, the majority of both WT and *Myd88^−/−^* mice had very high levels of IL-6, TNF-α, and IL-10, indicative of progressing sepsis (Fig. 4B to D). Also similar to the findings at 48 hpi, serum IFN-γ was largely absent in *Myd88^−/−^* mice (Fig. 4E).

Hematoxylin and eosin (H&E) staining of formalin-fixed livers at 72 hpi showed a small increase in inflammatory lesion severity in WT mice compared to that in *Myd88^−/−^* mice (Fig. 4F and G). These differences, while reproducible, were not significant. However, both groups of mice exhibited only mild to moderate liver pathology at 72 hpi; therefore, the data are suggestive of a potentially important role for MyD88 in the late stage of disease. In fact, *Myd88^−/−^* mice often appeared to be asymptomatic 12 h or less prior to death, whereas WT mice generally developed mild or moderate symptoms, suggesting that the acute phase of disease in the *Myd88^−/−^* mice occurs very rapidly (data not shown).

**FIG 5:**
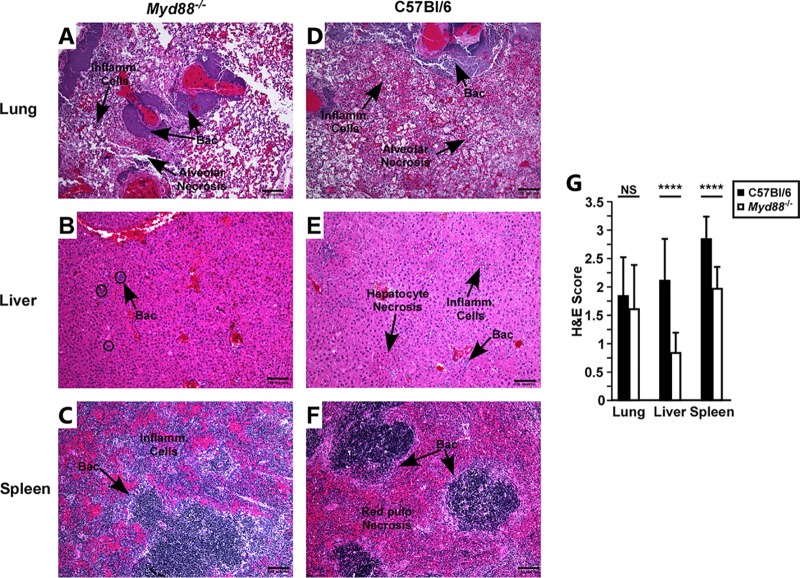
*Myd88^−/−^* mice that succumb to Y. pestis CO92 infection have reduced inflammatory foci and tissue necrosis. Lungs (A and D), livers (B and E), and spleens (C and F) from *Myd88^−/−^* (A to C) and C57BL/6 (D to F) mice that were challenged with Y. pestis CO92 and succumbed to disease were fixed in formalin and processed for histology. (A to F) Representative lesions; (G) severity scoring of inflammatory infiltrate and necrosis. Bars indicate standard deviations; data were collected in 3 independent trials (*n* = 14 WT mice, *n* = 20 *Myd88^−/−^* mice). Scale bar, 100 μm. Data were analyzed by an unpaired Student’s *t* test. ****, *P* < 0.0001; NS, not significant.

To determine whether moribund WT mice had increased pathology in the liver, we examined mice that succumbed to disease by histopathology. Although the pathological lesions in the lungs of *Myd88^−/−^* mice appeared similar to those of the WT mice in type and severity, inflammatory foci were small and infrequent in the liver in *Myd88^−/−^* mice, whereas in WT mice, multiple, sometimes large, inflammatory and necrotic foci were found in the liver (Fig. 5A to D). In all of the WT mice examined, the spleens had severe red pulp necrosis, whereas in *Myd88^−/−^* mice, there was reduced necrosis and increased immune cells in the spleen (Fig. 5E and F). In all tissues, bacteria were commonly visible by H&E staining. Severity scoring of the liver and spleen showed pronounced differences between WT and *Myd88^−/−^* mice at the end stage of plague, with significantly reduced severity in *Myd88^−/−^* mice (Fig. 5G). Together with the 72 hpi histopathology analyses, the data suggest that the inflammatory and necrotic lesions in the liver and spleen were due to the MyD88 response.

**FIG 6:**
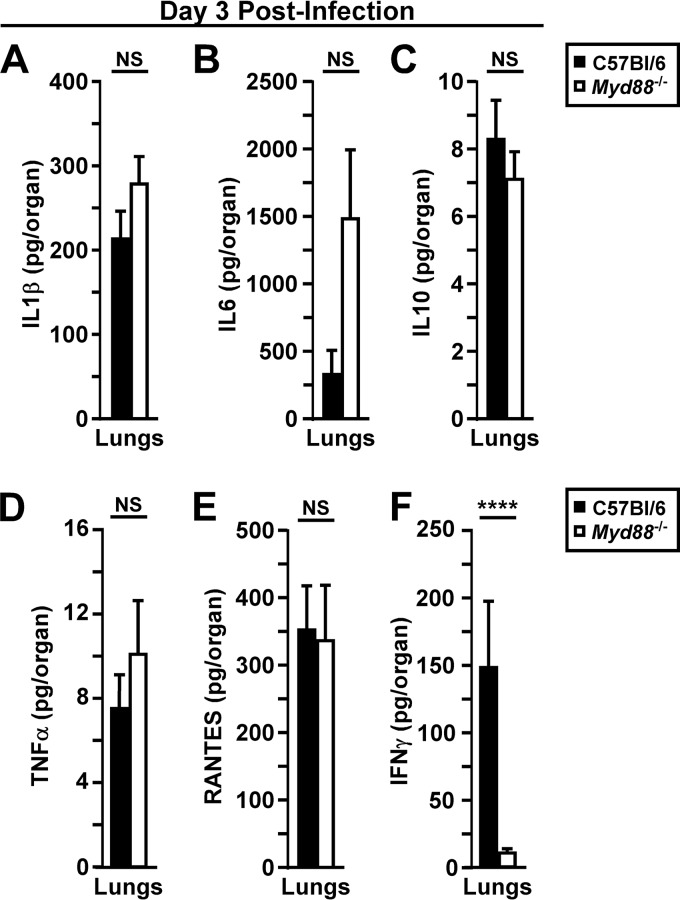
Pulmonary responses during disease phase are primarily MyD88 independent. Groups of 5 to 10 C57BL/6 or *Myd88^−/−^* mice were challenged by intranasal infection with 2,000 CFU of Y. pestis CO92. On day 3 postinfection, mice were euthanized, blood was collected, lungs were removed and homogenized in sterile PBS, and serum and lung homogenates were analyzed for the following cytokines by a Luminex multiplex assay: IL-1β (A), IL-6 (B), IL-10 (C), TNF-α (D), RANTES (E), and IFN-γ (F). *n* = 20 (B and D to F) or *n* = 10 (A and C) mice per group, with data collected in 2 to 4 independent trials. Bars represent standard errors. Data from all trials were combined and analyzed by the Mann-Whitney test. ****, *P* < 0.0001; NS, not significant.

Since the histopathology suggested that *Myd88^−/−^* mice recruit neutrophils to the lungs in the late stage of disease, we measured inflammatory cytokines, including IL-1β, IL-6, IL-10, TNF-α, and RANTES, in lung homogenates at 72 hpi. Contrary to the findings at 48 hpi, these cytokines were elevated in the lung homogenates of WT and *Myd88^−/−^* mice, with no detectable differences between the groups (Fig. 6A to E). In contrast, IFN-γ remained MyD88 dependent (Fig. 6F). Together, these results indicate that, unlike the case in early infection, MyD88-independent systemic inflammation is associated with lung pathology during the disease phase. Overall, these data suggest that sepsis and lethal secondary septicemic plague develop more rapidly in *Myd88^−/−^* mice.

## DISCUSSION

For many years, pulmonary infection by Y. pestis has been described as a biphasic inflammatory response driven by the T3SS, yet the underlying host factors that are targeted to mediate this response are not known (28). Here we demonstrated that MyD88 is at the center of this phenomenon. We showed that both phases of the biphasic inflammatory response involve MyD88. Very early MyD88-dependent inflammation was limited to KC, a neutrophil chemokine that we previously showed stimulates a protective neutrophil response during pulmonary Y. pestis infection. Indeed, at 18 hpi, *Myd88^−/−^* mice had increased bacterial titers in the lungs compared to WT mice. Yet this response allowed the escape of a small population of bacteria into a protected replicative niche. This event may be sufficient to trigger the immune suppressive phase. We think it likely that bacterial growth further stimulates MyD88-dependent inflammation that is ineffective. Overall, these data indicate a major role for MyD88 in the biphasic inflammatory response to Y. pestis. In contrast, the dissemination and subsequent development of secondary septicemic plague are not exacerbated by the MyD88 response, and in fact, it is protective against disseminated infection.

MyD88 is known to mediate signaling downstream of TLR and IL-1R family proteins, each able to activate the expression of pro- and anti-inflammatory cytokines (3). There are 13 TLRs and 11 IL-1R family members, some of which are antagonists that bind cytokines but lack the TIR domain. Although these proteins share the common ability to activate the formation of the myddosome, they serve distinct functions in cells and tissues. Tissue and inflammatory macrophages are primary targets of the Y. pestis T3SS *in vivo*, and alveolar macrophages become depleted in the early stage of infection (20, 23). The role of macrophage depletion has not been experimentally determined but is known to be caused by the bacterial T3SS. As T3SS effectors are known to stimulate inflammatory cell death, the prevailing model suggests that macrophage death, and the associated release of IL-1β and IL-18, is an important switch in stimulating the proinflammatory phase of the biphasic response.

As IL-1 and IL-18 receptors have TIR domains that activate MyD88, it is likely that the *Myd88^−/−^* mice have lost IL-18-dependent responses as well as IL-1-dependent responses. Loss of IL-18R signaling is known to reduce IFN-γ expression by NK cells in the lungs during bacterial infection; however, IFN-γ may not play a major role in defense against plague (29, 30). In addition, IL-18R activates expression of CXC chemokines such as KC (4). Consistent with the model described above, we observed coregulation of KC and IFN-γ in a MyD88-dependent manner at 48 hpi. Yet only KC was MyD88 dependent at 18 hpi. This observation suggests that the very early inflammatory response, though still MyD88 dependent, occurs through a distinct mechanism. In addition to inducing proinflammatory cytokines through NF-κB and mitogen-activated protein kinase (MAPK) signaling, IL-1 and IL-18 receptor signaling can also induce lipid mediators such as prostaglandin E2 (PGE2), which induces increased tissue-damaging nitric oxide (NO) production (31). Since *Myd88^−/−^* mice typically showed reduced tissue necrosis, PGE2 and/or other lipid mediators may also contribute to the pathological role of MyD88.

Therapeutic inhibition of the early MyD88 inflammatory response or its resulting damage to lung tissue would be expected to prevent development of bronchopneumonia. However, upon bacterial dissemination to secondary tissues, the inhibition of MyD88 would be detrimental, promoting bacterial replication and systemic disease. Anti-inflammatory treatments combined with antibiotics or other antibacterial treatment would be predicted to be synergistic in preventing the lethality of plague when given in the early stage. Recent studies in the murine bubonic plague model suggest that this is indeed the case (32). Corticosteroid treatment combined with neutralizing antibodies to Y. pestis improved survival. Importantly, in this study, the corticosteroid treatment reduced the recruitment of neutrophils to infected tissues yet bacterial growth was decreased. Consistent with the data reported here, these results emphasize the complexity of the host-pathogen interface during Y. pestis infection and the delicate balance necessary for an effective inflammatory response.

## MATERIALS AND METHODS

### Bacterial strains.

Yersinia pestis strains were routinely grown fresh from frozen stock by streaking for isolation onto heart infusion agar (HIA) plates supplemented with 0.005% (wt/vol) Congo red and 0.2% (wt/vol) galactose to verify retention of the pigmentation locus (33–36). For intranasal challenge studies, a single colony was used to inoculate heart infusion broth (HIB) supplemented with 2.5mM CaCl_2_ and grown for 18 to 24 h at 37°C and 125 rpm. All work with live, wild-type, *pgm*^+^ Y. pestis strain CO92 bacteria was performed in a select agent-authorized biosafety level 3 (BSL3) laboratory.

### Vertebrate animals.

All animal procedures were performed in compliance with the Office of Laboratory Animal Welfare and the National Institutes of Health *Guide for the Care and Use of Laboratory Animals* and were approved by the University of Missouri Animal Care and Use Committee.

C57BL/6J mice were the inbred strain background of the *MyD88^−/−^* mice (both strains obtained from Jackson Laboratories, Bar Harbor, ME). Mice were bred in-house at the University of Missouri. Male and female wild-type and mutant mice, ranging from 15 to 30 g in weight, were used for challenge experiments in approximately equal numbers. To model primary pneumonic plague, mice were challenged with a target dose of 3 to 6 LD_50_ (1,000 to 2,000 CFU) of Y. pestis CO92 by intranasal inoculation (37). The actual infection dose was verified by plating in all experiments. All infected mice were monitored by daily assignment of health scores, which involved assessments of their appearance and activity. Animals that survived to the end of the 14-day observation period or were identified as moribund (defined by pronounced neurologic signs, inactivity, and severe weakness) were euthanized by CO_2_ asphyxiation, followed by bilateral pneumothorax or cervical dislocation, methods approved by the American Veterinary Medical Association Guidelines on Euthanasia.

### Infection studies.

At the hours or days postinfection (hpi or dpi) indicated in the figure legends, infected mice were euthanized, and blood, along with the indicated tissues, was collected. Tissues were homogenized in sterile phosphate-buffered saline (PBS) and, along with blood, serially diluted and plated in duplicate on HIA for bacterial enumeration. After plating, serum was collected following centrifugation and, along with lung homogenates, treated with antibiotics to inactivate Y. pestis and then stored at −80°C until analysis. Alternatively, mice were euthanized and tissues were collected for histology. Tissues were fixed in 10% formalin for at least 48 h and then further processed for paraffin embedment and cut into 5-μm sections. Tissue sections were stained with hematoxylin and eosin, and coverslips were permanently affixed to stained slides. Sample identities were blinded for analysis. Severity scoring (0 to 3) was based on the size of necrotic and inflammatory lesions and the percentage of affected tissue, with a score of 3 representing the most severe. Serum and lung homogenate cytokines were measured by a Luminex multiplex assay (Sigma-Millipore, MO, USA) or by ELISA (PBL Assay Sciences, NJ, USA; R&D Systems, MN, USA).

### Statistical evaluation.

Data from all trials were combined and analyzed for statistical significance. Statistical significance was evaluated using Prism 7 (GraphPad Software, La Jolla, CA). Specific statistical evaluations are indicated in the figure legends; significance was concluded when the *P* was <0.05.

## Supplementary Material

Supplemental file 1
