# Supplementary material for: Yersinia pestis Exploits Early Activation of MyD88 for Growth in the Lungs during Pneumonic Plague
Source: Infect Immun. 2019 Mar 25;87(4):e00757-18. doi: 10.1128/IAI.00757-18 (PMC6434131; doi:10.1128/IAI.00757-18)
Supplement: Supplemental file 1 [file IAI.00757-18-s0001.pdf]

## Supplemental Figure S1.

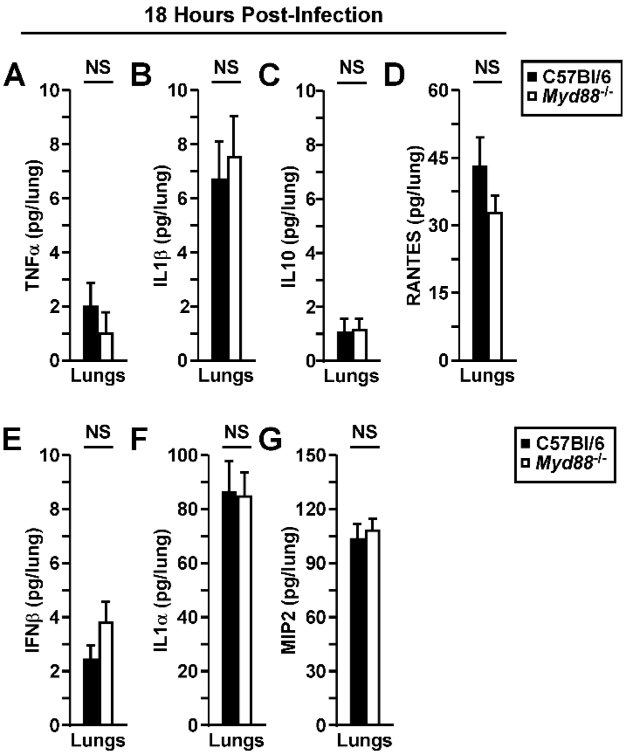


**Supplemental Figure S1.** *Suppression of inflammatory cytokine expression in the lungs in Myd88$^{-/-}$ and WT mice at 18 HPI.* Groups of 5 C57Bl/6 (filled bars) or *Myd88*$^{-/-}$ (open bars) mice were challenged by intranasal infection with 2,000 CFU *Y. pestis* CO92. After 18 hours of infection, mice were euthanized, lungs were collected for cytokine analyses: (A) TNFα, (B) IL1β, (C) IL10, (D) RANTES, (E) IFNβ, (F) IL1α, and (G) MIP2. Data were collected in 2 independent trials, n=10 per group. Bars represent standard error. Combined data were analyzed by t-test, NS: not significant.
